# Lifestyle Behavior Patterns and Socio-Demographic Predictors Among US College Students: Implications for Chronic Condition Risk and Public Health Interventions

**DOI:** 10.1177/15598276251357526

**Published:** 2025-07-03

**Authors:** Keegan T. Peterson, Melissa Bopp

**Affiliations:** 1 Department of Kinesiology, 311285 The Pennsylvania State University, University Park, PA, USA (KTP, MB)

**Keywords:** young adult, health promotion, preventive medicine, public health

## Abstract

**Purpose:** College students are uniquely positioned to develop healthy lifestyle behaviors that reduce chronic condition risk; however, disparities exist, and certain socio-demographic characteristics may impact behavior participation. **Methods:** Data were collected from a large, Northeastern US university from 2018–2024. Participants (n = 6197) self-reported their demographics, physical activity (PA: aerobic and muscle-strengthening (MS)), diet, sleep, and alcohol and substance use, which informed 6 latent class indicators: independently meeting aerobic and MS guidelines, meeting diet guidelines, obtaining frequent restful sleep, heavy alcohol use, and substance use. A four-class latent class model was deemed best for data fit. Regression covariates of sexual orientation, gender identity, certain racial/ethnic identities were included in models. **Results:** Four classes were identified: (1) healthy partygoer (36.8%), (2) balanced health seeker (9.6%), (3; referent) risky lifestyler (43.5%), (4) sedentary but health-conscious (10.0%). Identifying as a man, a sexual minority, and specific race/ethnicities significantly predicted class membership. **Discussion:** A moderate proportion of college students were classified as risky lifestylers and participated in health-diminishing behaviors over health-enhancing behaviors. Gender, sexual orientation, and race/ethnicity predicted membership in classes that may increase risk of chronic conditions, emphasizing the importance of tailored approaches to reduce threats to public health.

“Gender identity, sexual orientation, and race/ethnicity have been well-documented as predictors of negative health outcomes.”

Public health threats of physical inactivity,^1^ poor diet and nutrition,^2^ inadequate sleep,^3^ and heavy alcohol^4^ and substance use/misuse^5^ are increasing chronic conditions (e.g., obesity, heart disease, etc.) among adults, leading to high individual and social economic burden.^6^ These behaviors often cluster and have synergizing effects^7^; thus, identifying and examining patterns of lifestyle behaviors among certain at-risk populations (i.e., young adults), and how they vary by socio-demographic characteristics, remains paramount to address these threats. Without these understandings, our ability to tailor promotional efforts to reduce the current influx of chronic conditions experienced by US adults will remain stinted.

Young adulthood, often coinciding with college years, serves as a pivotal developmental period to develop lifelong habits. College student lifestyle behaviors of physical activity (PA), diet, sleep, and alcohol and substance use may be influenced by personal, social, and environmental changes through navigation of newfound independence, social engagements, and new living conditions.^8^ Because of these factors, it is essential to identify and examine lifestyle behavior patterns, given the opportunity of health promotion during higher education. Specific to PA behaviors, individuals are recommended to achieve 150 to 300 min per week of moderate-PA or 75 to 150 min per week of vigorous-PA, or an equivalent combination of both, and at least two days a week of muscle-strengthening activities.^1^ Aerobic activities support heart and lung function; while, muscle-strengthening can enhance strength and muscular endurance, emphasizing the need to participate in both activities.^1^ Importantly, college-attending individuals who are physically inactive during college are more likely to remain physically inactive post-graduation.^9^ Further, college students report unhealthy snacking, high-calorie convenience eating, reduced access to healthy foods, and infrequent fruits and vegetable consumption^10^; which may be associated with increased food insecurity and obesity rates.^11^ Reduced sleep quality and duration are also of concern,^12^ with academic and social pressures, irregular schedules, physical inactivity, and food insecurity being associated with poor sleep.^13^ The college environment can also increase exposure to alcohol and other substances, increasing the susceptibility of misuse among college students.^14^ This misuse may result in negative consequences of risky sexual behaviors, physical illness and injury, missed classes and reduced academic success, and altered cognition.^15,16^ These behavior patterns can vary by certain socio-demographic characteristics of gender identity, sexual orientation, and race/ethnicity, requiring improved tailored efforts to support health equity.

Specific to gender, college-attending women are less likely to meet PA guidelines,^17^ are more likely to make healthier diet choices,^18^ experience reduced sleep quality,^19^ and are less likely to misuse alcohol and other substances,^20^ when compared to men. Specific to race/ethnicity, minority students often report reduced rates of aerobic and muscle-strengthening (MS) activity participation,^17^ increased rates of unhealthy diets and food insecurity,^18^ poor sleep quality and duration,^21^ and are less likely to misuse alcohol and other substances,^20^ compared to non-Hispanic (NH) White students.^20^ Sexual minority (SM) college students are less likely to participate in aerobic and MS activities,^22^ report varying diet quality,^23^ reduced sleep quality,^24^ and more alcohol and substance use compared to heterosexual students.^20^ These disparities by socio-demographics remain pertinent when discussing health promotion efforts to support health equity among college students.

Latent class patterns have been previously mapped regarding similar lifestyle behaviors among US college students; however, there are limited studies that have included MS as an independent measure of PA, as well as studies assessing the role of multiple socio-demographic characteristics on class predictability. Thus, the aims of this latent class analysis were 2-fold: (1) uncover latent classes of lifestyle behaviors characterized by patterns of PA (aerobic and MS), diet, sleep, and alcohol and substance use among US college students and (2) investigate if socio-demographic characteristics of gender identity, sexual orientation, and race/ethnicity predict class membership.

## Methods

### Study Sample

Data for this study were collected at a large, Northeastern US university from 2018–2024 via an online, anonymous survey (Qualtrics, Provo, UT). The survey consisted of 50 questions documenting demographics, PA, diet, sleep, and alcohol and substance use. Participants were enrolled in general health and wellness courses and provided a link to an online survey at the beginning of each semester (Fall and Spring). This was not a required course assignment. The average completion rate across cohorts was 86%. An informed consent statement was provided at the beginning of the survey and participants could enter a drawing to receive a gift card upon survey completion. Analyses included 6197 participants. The university’s institutional review board approved this study.

### Measures

#### Physical Activity

The Global Physical Activity Questionnaire,^25^ assessed volume of moderate- (MPA) and vigorous- (VPA) intensity PA. Participants self-reported the number of days per week they engaged in MS activities. Weekly minutes of MPA and VPA were calculated through activity frequency and duration and were then multiplied by their corresponding metabolic equivalent of task (MET) value to calculate MET-mins/week (MPA = 4.0; VPA = 8.0). Participants were classified as meeting aerobic (≥600 MET-mins/week) and MS guidelines (≥2 days/week) based on current PA guidelines.^1^

#### Diet

Participants were provided the current US Dietary Guideline’s definitions for serving size amounts of fruits/vegetables and self-reported their average daily consumption. Participants who reported two or more servings of both fruits and vegetables per day were classified as meeting diet guidelines.^2^

#### Substance Use

Participants self-reported if they used any of the following substances in the past 30 days (yes/no): Cigarettes, Smokeless tobacco, and vaporizers and/or electronic cigarettes. Due to sample size, all substances were collapsed into one variable and classified based on use of at least one substance.

#### Alcohol Use

The Daily Drinking Questionnaire,^26^ assessed volume, quantity, and frequency of alcohol consumption. Participants were provided descriptions of standard drink equivalent of standard American beer, microbrew or European beer, single-serve wine and wine coolers, wine bottles, and hard liquor single-serve drinks and bottles. Participants self-reported the amount of alcohol they consumed during a typical week in the last 3 months from zero drinks to 10+ drinks per day. Participants were classified as heavy drinkers based on the National Institute of Alcohol Abuse and Alcoholism thresholds of ≥15 drinks/week for men and ≥8 drinks/week for women.^27^

#### Sleep

Participants were asked how many days during the past week they got enough sleep to feel well rested in the morning using a slider scale (0 = none; 7 = everyday). Participants were classified as obtaining frequent restful sleep (≥4 days) as used in a previous study among college students.^28^

#### Covariates

Participants self-reported their gender identity, sexual orientation, and race/ethnicity. Due to sample size, these variables were modified. Gender identity (man, woman, agender, androgyne, demigender, gender fluid, nonbinary, queer, questioning/unsure, trans man, trans woman, other, prefer not to disclose) was collapsed into two categories: Men [Man] and Women [Woman], with other identities being removed from analyses. Sexual orientation (straight/heterosexual, bisexual, gay, lesbian, pansexual, queer, questioning/unsure, other, prefer not to disclose) was collapsed into two categories: heterosexual [straight/heterosexual] and SM [all other orientations]. Full sexual orientation information is provided to promote transparency in SM health research (Table 1). Race/ethnicity was collapsed into 6 categories: NH White [White/Caucasian], Asian [Asian American], Hispanic/Latin(x) [Hispanic/Latin(x)], Black/African American [Black/African American], Multiracial [multiple identities], and Other [Other, Native American, Pacific Islander]. Participants who selected multiple races but also selected Hispanic/Latin(x) were coded as Hispanic/Latinx. For these variables to serve as numerical covariates, they were each re-coded as individual dummy variables (dichotomous; k-1 dummy variables) with the larger population serving as the reference group.^29^

**Table 1.** Sample Characteristics of US College Students (n = 6197).

	n (%)
Latent class indicators	
Met aerobic PA guidelines	
Yes	4799 (77.4%)
No	1398 (22.6%)
Met MS guidelines	
Yes	3277 (52.9%)
No	2920 (47.1%)
Met diet guidelines	
Yes	2932 (47.3%)
No	3265 (52.7%)
Obtained frequent restful sleep	
Yes	3378 (54.7%)
No	2810 (45.3%)
Heavy alcohol use	
Yes	1302 (21.0%)
No	4895 (79.0%)
Substance use	
Yes	1119 (18.1%)
No	5078 (81.9%)
Socio-demographic characteristics	
Gender identity	
Woman	3591 (57.9%)
Man	2414 (39.0%)
Sexual orientation	
Heterosexual	5302 (85.6%)
Bisexual	373 (6.0%)
Prefer not to disclose	154 (2.5%)
Questioning/unsure	101 (1.6%)
Gay	71 (1.1%)
Lesbian	68 (1.1%)
Pansexual	44 (0.7%)
Asexual	40 (0.6%)
Queer	34 (0.5%)
Other	4 (0.1%)
Same gender loving	3 (0.1%)
Race/ethnicity	
Non-Hispanic White	4453 (71.9%)
Asian	486 (7.8%)
Multiracial	357 (5.8%)
Other	323 (5.2%)
Hispanic/Latin(x)	319 (5.1%)
Black/African American	197 (3.2%)
Prefer not to disclose	62 (1.0%)

### Statistical Analysis

SAS 9.4 (SAS Institute, Cary, NC, USA) was used for analyses. Descriptive statistics describe the sample. Latent class analyses (LCA) identify similar subgroups within a larger heterogeneous sample based on multiple indicators^30^; which were conducted using the PROC LCA^29^ and LCABoostrap^31^ SAS macro programs. Six lifestyle behaviors were used as indicators: meeting aerobic guidelines, meeting MS guidelines, meeting diet guidelines, obtaining frequent restful sleep, heavy drinking, and substance use. LCA class fits ranged from one to six in a step-wise fashion, with the optimally fitted class being determined by the lowest Akaike information criteria (AIC), Bayesian information criteria (BIC), sample size adjusted BIC (ssaBIC) fit statistics, a bootstrap likelihood ratio test (BLRT), and item-response probabilities that support theoretical interpretability.^30^ Once the model was identified (aim 1), a regression model incorporated socio-demographic characteristics of gender identity, sexual orientation, and race/ethnicity to determine potential predictions of class membership (aim 2). Models yielded an odds ratio of the effects of each covariate on class membership. The reference latent class was the class with high probabilities of health-diminishing behaviors (i.e., heavy drinking, substance use) and low probabilities of health-enhancing behaviors (i.e., adequate PA, diet, sleep). Those identifying as a woman, heterosexual, and non-Hispanic White served as our referent group given their high frequency of occurring in the sample. A Bonferroni correction of *P* ≤ 0.01 accounted for multiple comparisons. We accepted the statistical significance of the odds ratio if the confidence interval did not include zero.

## Results

Overall, participants (n = 6197) were women (58.2%), heterosexual (85.6%), NH White (71.9%), and 20 ± 2 years of age. The majority met aerobic PA (77.4%) and MS guidelines (52.9%), did not meet dietary guidelines (52.7%), obtained frequent restful sleep (54.7%), and were not heavy alcohol drinkers (79.0%) or substance users (81.9%) (Table 1).

### Model Identification

Latent class analyses was used in an exploratory fashion to compare models with different latent classes to select the optimal solution describing patterns of lifestyle behaviors (Table 2). The BIC and ssaBIC values indicated a four-class model; however, the AIC indicated a five-class model.^30^ The BLRT (n = 99) was significant for each additional class added to the model but was nonsignificant once the five-class model was compared to the six-class model (Table 2). It remains common for parameter estimates to clearly indicate an optimal class solution; thus, theoretical interpretability and parsimony was considered.^30^ The four-class model was selected based on statistical criteria and pattern interpretability, and classes are distinguishable and can be individually labeled meaningfully.

**Table 2.** Model Fit Statistics for Latent Classes of Lifestyle Behaviors Among US College Students.

Class	LL	G^2^	*df*	AIC	BIC	ssaBIC	BLRT	E	Estimated Class Proportions	
1	−23739.74	1871.87	57	1883.87	1924.83	1905.76	—	—	—
2	−23110.47	613.32	50	639.32	728.05	686.74	0.01	0.55	0.45, 0.55
3	−22885.48	163.34	43	203.34	339.85	276.30	0.01	0.64	0.43, 0.09, 0.48
4	−22837.26	66.91	36	120.91	305.20	219.40	0.01	0.67	0.37, 0.10, 0.43, 0.10
5	−22826.79	45.98	29	113.98	346.05	238.01	0.01	0.53	0.13, 0.12, 0.24, 0.12, 0.38
6	−22820.14	32.67	22	114.67	394.2	264.23	0.20	0.57	0.13, 0.10, 0.16, 0.10, 0.11, 0.42

Abbreviations: LL, log-likelihood; df, degrees of freedom; AIC, Akaike information criteria; BIC, Bayesian information criteria; ssaBIC, sample size adjusted BIC; BLRT, bootstrap likelihood ratio test (n = 99); E, entropy.

Class membership probabilities and item-response probabilities described the classes of lifestyle behaviors (Table 3). Class one (36.8% of the sample) labeled “healthy partygoer” was comprised of students who had high/moderate engagement probabilities of all behaviors, including having met aerobic, MS, and diet guidelines, and obtained frequent restful sleep, but reported heavy drinking and used substances. Class two (9.6%) labeled “balanced health seeker” was comprised of students who reported a moderate likelihood to engage in most lifestyle behaviors, including having met aerobic, MS, and diet guidelines, obtained frequent restful sleep, and used substances, but are unlikely to drink heavily. Class three (43.5%) labeled “risky lifestyler” was comprised of individuals who had low engagement probabilities in any health-enhancing behaviors but had high engagement probabilities of health-diminishing behaviors, including not having met any aerobic, MS, and diet guidelines, not obtaining frequent restful sleep, but reported heavy drinking and substance use. Class four (10.0%) labeled “sedentary but health-conscious” was comprised of students who had moderate probabilities of most health-enhancing and -diminishing behaviors, including a moderate likelihood to have met diet guidelines, obtained frequent restful sleep, and heavily drank, but were unlikely to report having used substances or having met aerobic and MS guidelines.

**Table 3.** Item-Response Probabilities of Endorsing Lifestyle Behavior Indicators in Latent Class Among US College Students.

Indicator	Class 1	Class 2	Class 3	Class 4
	Healthy Partygoer (36.8%) (%)	Balanced Health Seeker (9.6%) (%)	Risky Lifestyler (43.5%) (%)	Sedentary but Health-Conscious (10.0%) (%)
Meeting aerobic PA guidelines	49.8	49.8	0.2	0.5
Meeting MS guidelines	83.9	75.7	20.0	9.7
Frequent restful sleep	51.1	57.8	37.4	48.0
Heavy alcohol use	99.9	23.2	81.6	48.4
Meeting diet guidelines	66.1	47.9	44.2	49.7
Substance use	88.3	54.5	99.8	7.4

Abbreviations: PA, physical activity; MS, muscle-strengthening.

### Latent Class Covariates

**Figure 1.**
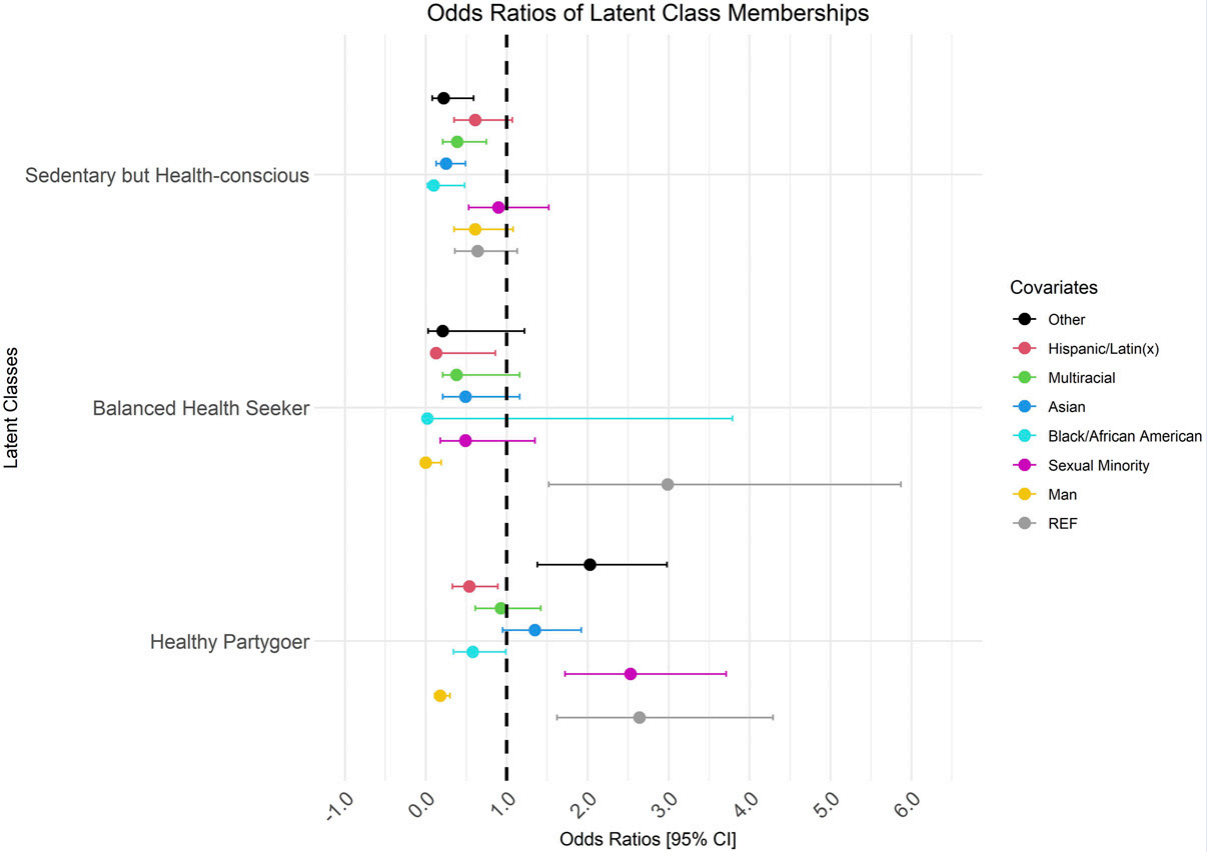
Odds ratios of latent class membership. Reference class (x = 1.0; class three, “risky lifestyler”). Abbreviations: REF, referent group; non-Hispanic White, heterosexual, woman. CI, confidence interval.

Table 4 describes the regression results; Figure 1 visualizes the results. Compared to a “risky lifestyler” (class three; reference class), the referent group [Woman, heterosexual, NH White] was associated with 164% greater odds of being a “healthy partygoer” (class one), 199% greater odds of being a “balanced health seeker” (class two). Although nonsignificant, women were associated with 36% lower odds of being “sedentary but health-conscious” (class four).

**Table 4.** Gender Identity, Sexual Orientation, and Race/Ethnicity as Predictors of Membership in Latent Classes of Lifestyle Behaviors Among US College Students.

	Class 1	Class 2	Class 3	Class 4
	Healthy Partygoer (36.8%)	Balanced Health Seeker (9.6%)	Risky Lifestyler (43.5%)	Sedentary but Health-Conscious (10.0%)
Intercept [referent = non-hispanic White, heterosexual, woman]			REF	
β_0_	0.97	1.10		−0.45
Odds ratio [95% CI]	2.64 [1.62, 4.29]	2.99 [1.52, 5.87]		0.64 [0.36, 1.13]
Man***				
β_1_	−1.71	−5.39		−0.49
Odds ratio [95% CI]	0.18 [0.11, 0.30]	0.00 [0.00, 0.19]		0.61 [0.35, 1.08]
Sexual minority***				
β_2_	0.93	−0.72		−0.11
Odds ratio [95% CI]	2.53 [1.72, 3.71]	0.49 [0.18, 1.35]		0.90 [0.53, 1.52]
Black/African American***				
β_3_	−0.54	−3.79		−2.30
Odds ratio [95% CI]	0.58 [0.34, 0.99]	0.02 [0.00, 3.79]		0.10 [0.02, 0.48]
Asian***				
β_4_	0.30	−0.71		−2.30
Odds ratio [95% CI]	1.35 [0.95, 1.92]	0.49 [0.21, 1.16]		0.25 [0.13, 0.49]
Multiracial**				
β_5_	−0.07	−0.97		−0.94
Odds ratio [95% CI]	0.93 [0.61, 1.42]	0.38 [0.13, 1.08]		0.39 [0.21, 0.75]
Hispanic/Latin(x)				
β_6_	−0.61	−2.03		−0.49
Odds ratio [95% CI]	0.54 [0.33, 0.89]	0.13 [0.02, 0.86]		0.61 [0.35, 1.07]
Other race/ethnicity***				
B_7_	0.71	−1.56		−1.52
Odds ratio [95% CI]	2.03 [1.38, 2.98]	0.21 [0.03, 1.22]		0.22 [0.08, 0.59]
LL = −21321.35				

Abbreviations: CI, confidence interval; LL, log-likelihood.

Model significance **P < .01, ***P < .001.

Men, compared to women, were significantly associated with 82% lower odds of being in class one and the same odds of being in class two, compared to class three. Although nonsignificant, men were associated with 39% lower odds of being in class four. SM identity, compared to heterosexual identity, was associated with 153% greater odds of being in class one, compared to class three. Although nonsignificant, SM identity was associated with 51% lower odds of being in class two and 10% lower odds of being in class four, compared to class three. Black/African American identity, compared to all other race/ethnicities, was associated with 42% lower odds of being in class one and 90% lower odds of being in class four, compared to class three. Although nonsignificant, Black/African American identity were associated with 98% lower odds of being in class two, compared to class three. Asian identity, compared to all other race/ethnicities, was significantly associated with 75% lower odds of being in class four, compared to class three. Although nonsignificant, Asian identity was associated with 35% greater odds of being in class one and 51% lower odds of being in class two, compared to class three. Multiracial identity, compared to all other races/ethnicities, was significantly associated with 61% lower odds of being in class four, compared to class three. Although nonsignificant, multiracial identity was associated with 7% lower odds of being in class one and 62% lower odds of being in class two, compared to class three. Other [Native American, Pacific Islander, Other] identity, compared to all other race/ethnicities, was associated with 103% higher odds of being in class one and 78% lower odds of being in class four, compared to class three. Although nonsignificant, Other identity was associated with 79% lower odds of being in class two compared to class three. Hispanic/Latin(x) identity was not a significant covariate in the model.

## Discussion

Overall, these findings suggest that US college students who participated in this study engage in similar lifestyle behaviors, resulting in four meaningful classes based on aerobic and MS PA, diet, sleep, and alcohol and substance use behaviors. Given the vulnerable period of habit development during college, it remains paramount to identify and examine these patterns to ensure health behavior participation can transcend into later life. Gender identity, sexual orientation, and race/ethnicity have been well-documented as predictors of negative health outcomes; thus, this study provides novel findings pertaining to lifestyle behavior profile predictability among historically minoritized groups to support tailored health promotion efforts. The patterns identified ranged from high probabilities of engaging in both health-enhancing (i.e., PA, diet, sleep) and health-diminishing behaviors (i.e., alcohol/substance use), to high probabilities of engaging in health-diminishing behaviors and low probabilities of engaging in health-enhancing behaviors. This research confirmed that health behaviors often interact and cluster together,^7^ and that social and cultural factors dictate behavior participation among college students.^8^

As discussed, latent class model selection within the social sciences relies on parameter estimates but can also be informed by previous research.^30^ This was supported through the identification of class one, “healthy partygoer,” which described students who report high/moderate probabilities of having met all health-enhancing guidelines of PA, diet, and sleep, but also report high probabilities of health-diminishing behaviors like having drank heavily and used substances. This pattern is similar to research among college students that identified over 60% of college students meet PA guidelines^32^; albeit, this review (k = 432) included 31 studies that assessed both aerobic and MS activity, and only 2 studies that only included MS activity. The current study’s inclusion of meeting MS guidelines as an independent variable provides much-needed evidence regarding MS among college students, especially regarding clustering of PA behaviors. This work also supports findings that increased PA is related to sleep quality,^33^ a higher likelihood to binge drink, and reduced likelihood to use tobacco/smoke.^34,35^ The college social environment may explain participation in both health-enhancing and health-diminishing behaviors, given that peer groups and networks may predict adolescent behavior.^8^

Specifically, compared to class one, class two, “balanced health seeker,” are moderately likely to have engaged in all behaviors although they are less likely to have reported heavy drinking and substance use. Interestingly, both classes were comprised of individuals who were more likely to have met MS guidelines compared to aerobic guidelines, which may provide novel findings to PA participation among college students. Class three, “sedentary but health-conscious” is similar to class two given these students reported healthy diets and sleep, and moderate amounts of alcohol and substance use, but differs by a stark decline in participation in aerobic and MS activities. In contrast, class three “risky lifestyler,” the largest proportion of students, reported participating in only health-diminishing behaviors of heavy drinking and substance use, which, is alarming given the key developmental period of habit forming while attending college. Classes three and four highlight the 40% of college students who are not meeting PA guidelines,^32^ and engage in health-diminishing behaviors that are common among college-attending individuals.^14,16^ Also, the high likelihood of substance use in class one and three supports the recent influx of students using e-cigarettes/vaporizers, and the increased likelihood of initiating cigarette/tobacco use.^36^ Young adults are experiencing cardiometabolic risk factors that are increasing their likelihood of later-life cardiovascular disease,^37^ and these findings may add necessary contexts.

Importantly, identifying as a man, a SM, and Black/African American, Asian, Multiracial, or Other [Native American, Pacific Islander, Other] significantly predicted membership in lifestyle behavior classes that may increase the risk of chronic conditions. Men had lower odds of being in class one when compared to class three and the same odds of being in class two, demonstrating their affinity for participating in worse health behaviors but a potential balance between health-enhancing and -diminishing behaviors. This is similar to previous research which found women are less likely to drink heavily or use substances,^20^ but in contrast to research highlighting men are more likely to be physically active and sleep well.^17,19^ This is also demonstrated by our referent group having an affinity for participating in healthier behaviors. Albeit, high likelihood of reporting heavy drinking and substance use was evident in class one, which is supported by recent research describing a narrowing gap between men and women who drink heavily,^38^ and that social and environmental contexts may explain substance use.^39^

Similarly, SMs had higher odds of being in class one when compared to class three, demonstrating that are more likely to participate in both health-enhancing and -diminishing behaviors. Trends of these individuals having lower odds of class two and four may be supported by previous research reporting SM students are less likely than their peers to participate in PA (aerobic and MS),^22^ and higher odds of being in class one may be supported by findings detailing that SMs are more likely to report binge drinking and use substances compared to their peers.^20^ Describing these odds remains complex given the limited availability of research examining these behaviors among SM college students; however, these findings elucidate behavior patterns among SMs during a key developmental period and support improved tailored promotional efforts.

Regarding racial/ethnic identities, Black/African American, Asian, Multiracial, and Other had lower odds of being in class four when compared to class three, noting they reported participation in worse health behaviors. Black/African American identity was also associated with lower odds of being in class one, while Other identity was associated with higher odds. This supports literature detailing reduced participation in health-enhancing behaviors (i.e., healthy diet, adequate sleep, being active),^17,18,21^ and may highlight social and cultural difficulties adopting and adhering to healthy behaviors or adjusting to a predominantly White institution. Uniquely, Other identity individuals had higher odds of being in class one, highlighting their high likelihood of participating in both health-enhancing and -diminishing behaviors. This may be explained by Pacific Islander men being more likely to meet MS guidelines when compared to NH White men; although the opposite was true for women.^17^ This classification is also in contrast with previous findings that racially/ethnically diverse students often report less alcohol and substance use, compared to NH White students.^20^ Previous research has identified social environments may dictate behaviors,^8^ which may be supported by the large population of NH White students at this university. These findings add to the complexity of behavior participation among multiple races/ethnicities and how factors influencing health behaviors may vary, whether helpful or harmful, but highlight a need for improved tailored health promotion efforts.

This study supports health behavior research among young adults, a group that is often not considered at risk for chronic conditions, and successfully mapped behavioral patterns that depict many students do not participate in adequate health-enhancing behaviors of PA, a healthy diet, and adequate sleep. The strength of this study lie within it is inclusion of separate measures of physical activity, both aerobic and MS, and the added findings of the role certain socio-demographics may play in class membership of lifestyle behaviors among US college students. The findings highlight that gender, sexual orientation, and race/ethnicity play a significant role in lifestyle behavior participation among college students but also highlight similarities between heterogeneous groups. College lifestyle interventions are lackluster,^40^ and students will continue to be at risk of developing later-life chronic conditions without improved interventions.

This study also has limitations. Our sample was mostly NH White, women, and heterosexual, reflective of a large northeastern US university; however, the findings remain impactful when discussing population trends on college campuses. The self-report nature of our measures from students enrolled in general health and wellness classes may provide overestimates of a more active sub-population; however, these findings assist in understanding which groups may be more inclined to participate in health-enhancing or -diminishing behaviors. Future studies should use longitudinal, objective measures to determine potential shifts in behaviors as students’ progress through their college education; while accounting for socio-demographic influences.

## Conclusion

Understanding health behaviors among young adults remains paramount to reducing later-life chronic disease conditions and associated individual and societal economic burden. This study found that a moderate proportion of college students participate in only health-diminishing behaviors (i.e., heavy drinking and substance use), with smaller proportions engaging in both health-enhancing behaviors (i.e., PA, diet, sleep) and -diminishing behaviors. Gender identity, sexual orientation, and race/ethnicity were significant predictors of class membership. This pattern mapping is required to understand and promote health during a formative period in development, emphasizing tailored college health promotion efforts to promote health equity. Without improved approaches, current public health threats will continue to plague the US population.

## Data Availability

Data can be made available upon request.*
